# Dielectric properties of semi-insulating Fe-doped InP in the terahertz spectral region

**DOI:** 10.1038/s41598-017-07164-1

**Published:** 2017-08-04

**Authors:** L. N. Alyabyeva, E. S. Zhukova, M. A. Belkin, B. P. Gorshunov

**Affiliations:** 1 Moscow Institute of Physics and Technology, Dolgoprudny, Moscow Region 141700 Russia; 2 A.M. Prokhorov General Physics Institute, Russian Academy of Sciences, Moscow, 119991 Russia; 3 Department of Electrical and Computer Engineering, The University of Texas at Austin, Austin, TX 78712 United States of America

## Abstract

We report the values and the spectral dependence of the real and imaginary parts of the dielectric permittivity of semi-insulating Fe-doped InP crystalline wafers in the 2–700 cm^−1^ (0.06–21 THz) spectral region at room temperature. The data shows a number of absorption bands that are assigned to one- and two-phonon and impurity-related absorption processes. Unlike the previous studies of undoped or low-doped InP material, our data unveil the dielectric properties of InP that are not screened by strong free-carrier absorption and will be useful for designing a wide variety of InP-based electronic and photonic devices operating in the terahertz spectral range.

## Introduction

Semi-insulating (SI) iron-doped indium phosphide (InP:Fe) is widely used in electronic and photonic devices operating in the terahertz spectral range (THz range, 0.1~10 THz), including Schottky diode detectors^1^, high-electron mobility transistors^2^, photomixers^3^, and quantum cascade lasers (QCLs)^4–6^. Nominally updoped InP crystals always contain different unintentional impurities due to the growth processes with the concentrations up to 5·10^15^ cm^−3^ that results in shallow donor or acceptor energy levels within the energy gap. Iron doping provides acceptor levels in the mid-gap region of InP that compensate residual shallow donors and produce material with virtually no free carriers^7^. High-resistivity semiconductors are highly desired for devices operating in the THz spectral range as the optical losses at THz frequencies are typically dominated by the free-carrier absorption.

Given the importance of InP material to photonics and electronics, its optical properties have been extensively studied across the electromagnetic spectrum^8–17^. However, THz optical properties of SI InP have not been reported in the literature yet. The dielectric constants of nominally undoped and low-doped InP have been studied in the THz spectral range recently^10, 11^; however, they are strongly affected by a free carrier plasma that screens the intrinsic characteristics of SI InP material, especially at frequencies below 5 THz.

In this study, we present detailed investigation of the electrodynamic response of SI InP:Fe in the 2–700 cm^−1^ (0.06–21 THz) spectral region. The studied samples are obtained from commercial vendors and have resistivities exceeding 5 × 10^6^ Ω·cm that corresponds to a free-carrier concentration below 10^9^ cm^−3^. As expected for such low free carrier concentration, our optical data show no plasma effect in the entire spectral range of interest. Given the importance of SI InP to a variety of applications^1–6^, we believe that this report will be useful to a wide range of groups involved in the microwave and THz semiconductor devices research and development.

## Experimental details

### Materials

Two semi-insulating InP:Fe wafers with the nominal thicknesses of 350 ± 25 and 1000 ± 25 μm, obtained from two different vendors (AXT and Wafer Technology Ltd., respectively) were studied. Approximately 1 × 1 cm^2^ square sections were cut from the wafers and the thicknesses of the squares were measured at multiple locations to be 360 ± 2 μm and 991 ± 2 μm. The difference in the dielectric data obtained for the two samples was within the measurement error. Below we present and discuss the data obtained with the thicker sample.

### Experimental setup and data processing

The measurements of the dielectric properties in the *ν* = 0.21–3.00 THz range (7–100 cm^−1^) were performed using a pulsed THz TeraView TPS-Spectra-3000 time-domain spectrometer. A spectrometer based on monochromatic and frequency-tunable continuous-wave (CW) backward-wave oscillators^18^ was used for the measurements in the 0.06–0.30 THz range (2–10 cm^−1^). Both spectrometers provide a possibility to derive the spectra of dielectric parameters - complex permittivity, dynamic conductivity, refractive index, etc., *directly* by measuring the values of the amplitude and the phase of the electric field of the radiation/electromagnetic wave passed through the plane-parallel sample. Additionally, we have performed high resolution (Δ*ν* < 0.3 cm^−1^) measurements of transmission and reflection coefficients of our samples in the 0.9–21 THz range (30–700 cm^−1^) using a standard vacuum Fourier-transform infrared spectrometer (FTIR) (Bruker Optics Vertex 80 v). For the data shown in the figures, we combined the data obtained with the backward-wave oscillators in the 2–10 cm^−1^ range with the data obtained by the TeraView system in the 10–100 cm^−1^ and the data obtained by the FTIR in the 100–700 cm^−1^ range. The results obtained by different measurement techniques in the overlapping spectral regions were the same within the experimental uncertainties.

Due to low absorption, the spectra of the transmission and reflection coefficients of our samples contain pronounced maxima and minima that arise due to the interference of the radiation within plane-parallel slabs (a well-known Fabry-Perot effect^19, 20^). These spectra allow for the most precise determination of the dielectric parameters of the samples at the frequencies of the interference maxima where the interaction of the probing radiation with the material is the most effective. This approach was used in the present research: the dielectric parameters of the studied InP:Fe samples were determined by modeling the measured transmission coefficient maxima based on the Fresnel expressions that describe the optical properties of a plane-parallel layers. At frequencies where the samples were not transparent due to strong phonon or impurity absorption resonances, the dielectric parameters of the samples were determined by simultaneous processing both transmission and reflection spectra. To get the parameters of each resonance, we have modeled them with the Lorentzian lineshapes:

1 $${\varepsilon }^{\ast }(\nu )=\varepsilon \text{'}(\nu )+i\varepsilon \text{'}\text{'}(\nu )=\sum _{j}\frac{{f}_{j}}{({\nu }_{j}^{2}-{\nu }^{2})+i{\nu }_{j}{\gamma }_{j}}$$

Here *ε*ʹ(*ν*) = *n* ^2^(*ν*) − *κ* ^2^(ν) and *ε*″(*ν*) = 2*nκ* are the real and imaginary parts of the complex dielectric permittivity *ε* ^*^(*ν*) = *ε*ʹ(*ν*) + i*ε*″(*ν*), *n* is the real and *κ* is the imaginary part of the complex refractive index n* = *n* + i*κ*, *f*_*j*_ = Δ*ε*_*j*_ *ν*_*j*_^2^ is the oscillator strength of the *j*-th resonance, Δ*ε*_*j*_ is its dielectric contribution, *ν*_j_ represents the resonance frequency, and *γ*_*j*_ is the damping factor.

## Results and Discussion

**Figure 1:**
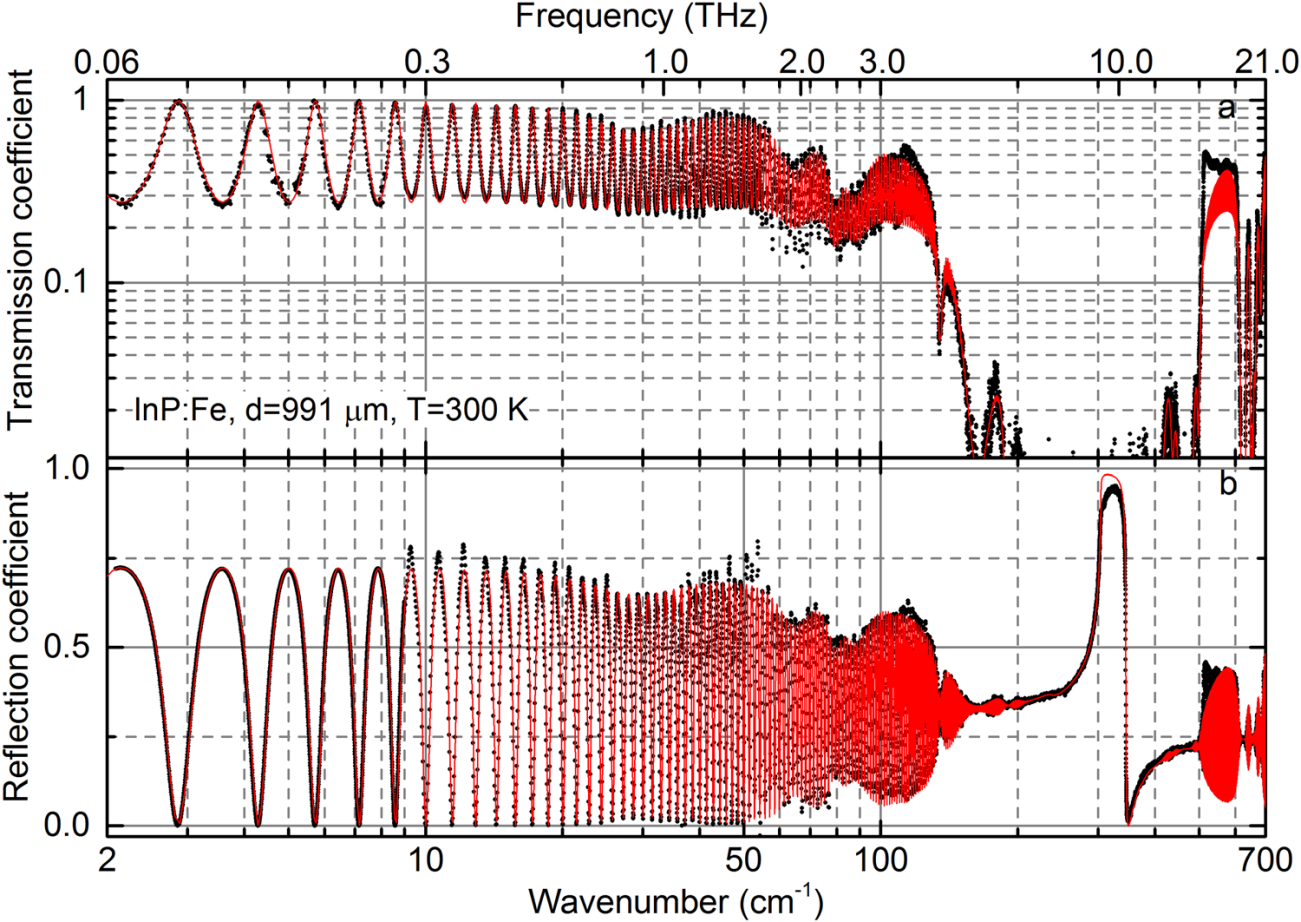
Room-temperature transmission (**a**) and reflection (**b**) spectra of semi-insulated Fe-doped InP wafer of 991 µm thickness. Black dots are the experimental data, red solid lines present theoretical modeling described in the main text.

The combined transmission and reflection spectra are shown in Fig. 1 (dots), together with the spectral modeling results (lines). Below 150–200 cm^−1^ the interferometric Fabry-Perot oscillations are clearly resolved with the maxima in the transmission spectrum corresponding to the minima in reflectivity. The interferometric effect is also seen at frequencies above 500 cm^−1^ where the absorption of the crystal is not too strong. A number of absorption bands are observed as pronounced minima in the transmission spectrum above 100 cm^−1^ and weaker absorption features are seen at lower frequencies. The origin of these absorption peaks is discussed below.

**Figure 2:**
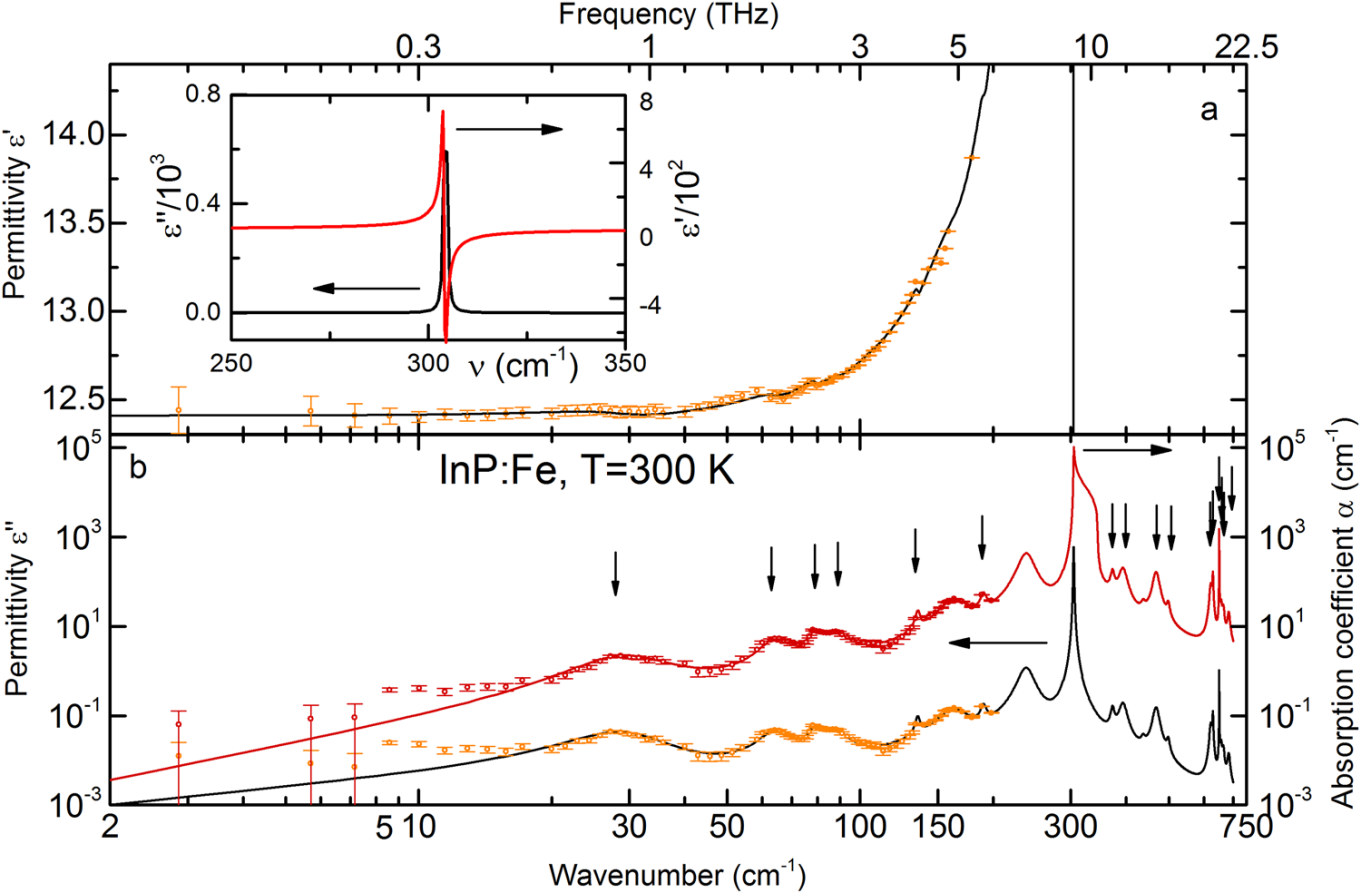
Room temperature terahertz-infrared spectra of the real (**a**) and imaginary (left vertical axis in panel (**b**)) parts of the dielectric permittivity and of the absorption coefficient *α* (right vertical axis in panel (**b**)) of Fe-doped InP. Dots are the experimental data, solid lines are the modeling curves described in the main text. The weak absorption lines assigned to multi-phonon interaction are designated with vertical arrows in panel b. The key parameters of the absorption lines are presented in Table 1 (cf. Eq. (1)). The inset in panel (**a**) shows the dielectric permittivity near the transverse optical phonon resonance.

Presented in Fig. 2 are the broad-band spectra of the real and imaginary dielectric permittivities and of the absorption coefficient of SI InP:Fe. The absorption coefficient was determined as $\alpha =\frac{\omega }{c}Im(\sqrt{\varepsilon ^{\prime} +i\varepsilon ^{\prime\prime} })$, where ω is the circular radiation frequency and c is the speed of light. The data reveals a complex structure with a set of absorption lines with the most intensive one located at 304 cm^−1^ (see Table 1). The strongest absorption line shown separately in the inset is a well-known transverse optical (TO) phonon^21^. This mode is responsible for the low transmission values between ≈150 cm^−1^ and ≈500 cm^−1^ in Fig. 1a and for the corresponding characteristic dispersion in the reflectivity spectrum in Fig. 1b. There might be weaker absorption bands in the frequency interval of the reststrahlen band, 300–350 cm^−1^ that are, however, not resolved in the spectra due to the dominant effect of the TO phonon absorption. Less intensive absorption bands on the left and right wings of the phonon resonance are assigned to multi-phonon (summation or differential^22^) processes (indicated in Fig. 2b with vertical arrows) or to the electron/hole transitions involving impurities.

**Table 1:** Parameters of the absorption lines (Eq. (1) in the text) identified in the dielectric data from the SI InP crystals.

This work					Literature	Assignment
	Δε (±)	γ (±), (cm^−1^)	f (±) (cm^−2^)	ν (±) (cm^−1^)	*ν* (±) (cm^−1^)	
1	0.018 (0.001)	14.5 (1.5)	16 (3)	29.3 (1.0)	25 (10)	LO-TO(L)
					28	LO-TO1(hex)
2	0.006 (0.002)	11.5 (2.5)	24 (7)	63.4 (1.0)	62	Si, S
					58	LA-TA2(hex)
3	0.003 (0.002)	8.4 (2.8)	16 (10)	79.3 (0.3)	89	LA-TA1(hex)
4	0.004 (0.001)	11.0 (2.2)	30 (10)	87.8 (0.3)	113 (4)	LA-TA(L)
5	0.0014 (0.0005)	3.6 (1.9)	26 (7)	135.1 (0.1)	132	2TA1(hex)
					135 (6)	2TA(X)
6	0.019 (0.001)	29 (3)	500 (30)	162.4 (0.7)	169	Ge
7	0.004 (0.001)	7 (1)	140 40)	190.0 (0.9)	191	LO-LA(hex)
					194	2TA2(hex)
8	0.128 (0.065)	22.0 (4.8)	7900 (4700)	240 (15)	240	Zn, Si, Be, Mg, Mn
9	2.714 (0.015)	0.7 (0.3)	252800 (3500)	304.2 (0.2)	303 (7)	TO(Г)
10	0.008 (0.007)	9.4 (7.4)	650 (570)	370 (20)	372 (6)	TO + TA(L)
11	0.011 (0.005)	18.7 4.1)	1700 (900)	394.2 (7.5)	392 (10)	TO + TA(X)
					395 (11)	TA + LO(L)
12	0.007 0.003)	14.2 (5.0)	1500 (600)	468 (0.5)	467	LA + TO(hex)
13	0.0002 (0.0001)	9.0 (4.5)	50 (25)	497.8 (1.8)	483 (8)	LA + TO(L)
					504	LA + TA1(hex)
14	0.0011 (0.0002)	11.9 (2.1)	430 (80)	624.4 (1.3)	614 (14)	2TO(Г)
15	0.0003 (0.0002)	3.2 (0.8)	120 (80)	627.6 (1.4)	634 (20)	2TO(L)
16	0.003 (0.002)	1.5 (0.8)	900 (600)	650.3 (0.2)	648 (14)	2TO(X)
					648	LO + TO(Г)
17	0.00020 (0.00005)	8.5 (1.6)	87 (1)	656.9 (0.5)	656 (10)	TO + LO(X)
18	0.00020 (0.00005)	8.4 (1.2)	80 (1)	663.6 (0.6)	657 (15)	TO + LO(L)
19	0.00015 (0.00005)	12.1 (3.2)	80 (1)	683.1 (0.5)	680 (20)	2LO(L)

The notations in the table are as follows: Δε – dielectric strength, γ – damping, f – oscillator strength, *ν* - resonance frequency. The “Literature” and “Assignment” columns refer to the literature values of the resonance frequencies and our assignment based on the literature. Phonon coupling assignments are done according to Koteles^26^. The designations in brackets refer to phonon branches locations: “hex” refers to a location on the (111) hexagonal face of the Brillouin zone boundary in accordance with^26^, Γ is the center of the Brilloiun zone (000), L is the center of the hexagonal face in the (111) wavevector direction, and X is the center of the cubic face in the (100) wavevector direction. Impurity assignments are done according to refs 23, 24.

Nominally pure InP crystals contain different unintentional impurities due to the growth processes with the concentrations up to 5·10^15^ cm^−3^. The most common unintentional impurities in the SI InP are Si, S, Zn, C^15, 17^. In order to trap the free carriers produced by these impurities, InP crystals are doped with iron that creates acceptor levels in the mid-gap region of InP^7^. The activation energy of Fe impurity is 640 meV^16^ (frequencies above 5100 cm^−1^) and it is not expected to affect our measured spectra. The energies of the transitions related to the defects, i.e. indium and phosphorus vacancies and antisites are higher than 100 meV^16^ (frequencies above 800 cm^−1^), and are also not expected to show up in our spectra. However, the ionization energies of shallow donors (Si, S, Sn, Ge) are about 7.65 meV^23^, and of shallow acceptors (Zn, C, Si, Mn, Be, Mg) are about 25–40 meV^13, 24^. These transitions are likely to produce absorption features seen in our spectra.

The lowest-frequency absorption band at about 30 cm^−1^ in the spectra in Fig. 2 was previously observed in ref. 25. In accordance with the temperature dependence of the oscillator strength, this band was associated with the two-phonon absorption; however, the authors in ref. 23. did not assign the line to a specific phonon type and location. Koteles and Datars^26^ predicted an absorption line associated with the differential LO-TO phonon absorption that should be located either at the L or “hex” (see below) points of the Brillouin zone and that is expected to appear in the region 15–35 cm^−1^. The authors of ref. 26. also observed a number of two phonon summation and differential absorption lines which they attributed to phonons located at the X point, L point, and a point, located somewhere on the (111) hexagonal face of the Brillouin zone boundary, designated as “hex”.

The next absorption band at about 64 cm^−1^ could be a manifestation of differential phonon absorption LA-TA2 (hex)^24^ and/or an impurity transition. The energy of the residual donor impurities is reported to be about 7.65 meV (61.7 cm^−1^)^23^. The small difference between the observed location of the band and the literature data of the shallow donor impurity transition can be explained by the dependence of the energy gap of InP on the donor content and by the mutual influence of several impurities presented within its crystal lattice.

The two lines observed at 79 and 88 cm^−1^ could also be attributed to a differential phonon absorption. According to the data in ref. 26, the phonon positions are determined with an uncertainty of ±10 cm^−1^ which explains the difference between the peak positions obtained in this study and in the literature (see Table 1).

The line at 163 cm^−1^ is apparently some acceptor impurity contribution. It is unlikely that this line is a manifestation of an absorption due to a two-phonon or a compensated shallow donor process, since its dielectric contribution and oscillator strength are about an order of magnitude higher than those of the neighboring lines. According to the literature data^24^, Ge impurity has the ionization energy of 21 meV and could correspond to the line at about 163 cm^−1^.

At 235 cm^−1^ (29.1 meV), a relatively strong absorption band is registered. Due to its high intensity (oscillator strength), it is unlikely to be attributed to a multi-phonon absorption process. This line may be a manifestation of a shallow acceptor. Taking into account the position of the band, the most possible impurities could be Mn, Si, Be or Mg. We note that this line may also be produced by Zn contamination.

**Figure 3:**
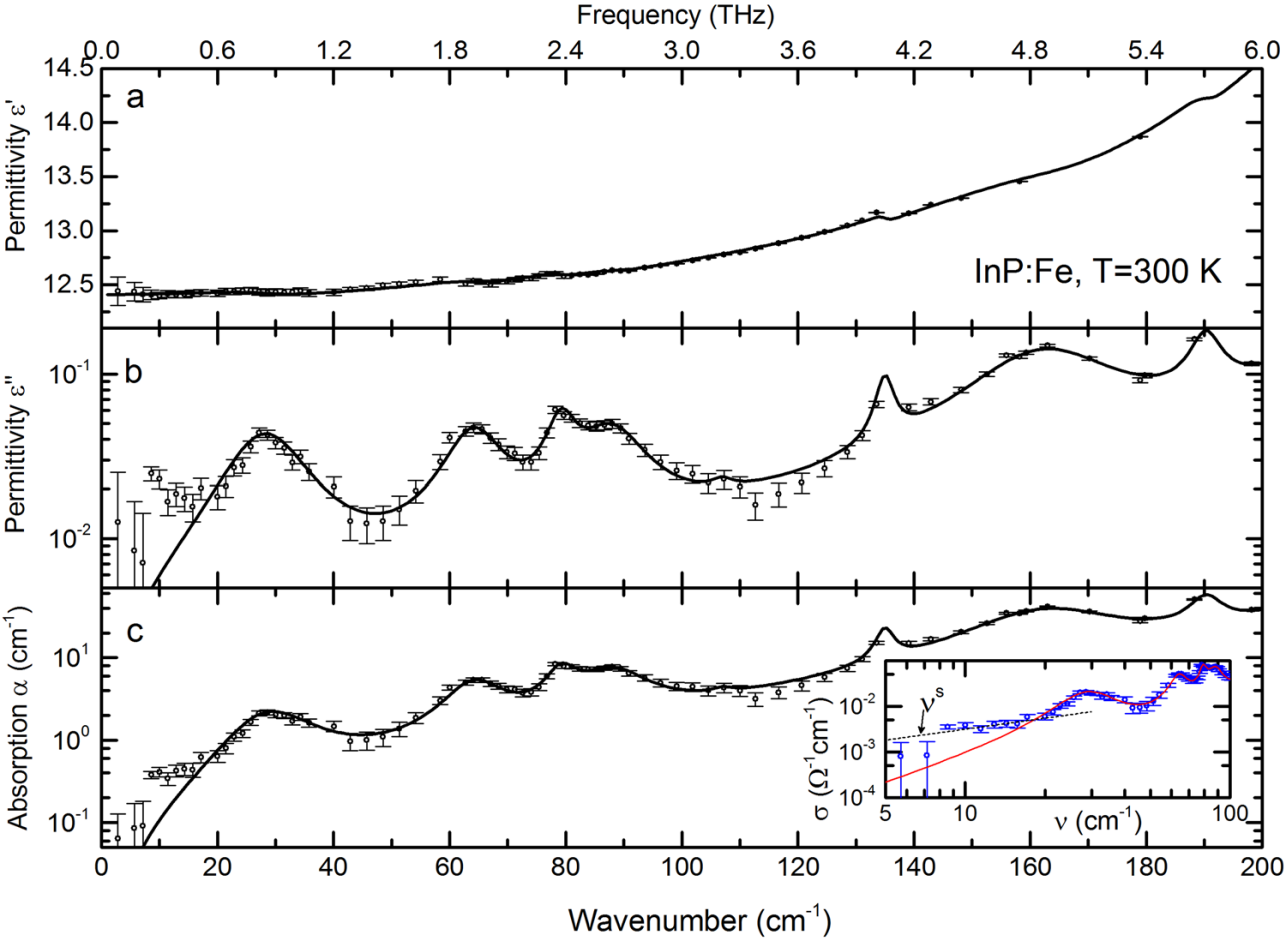
Room temperature low-frequency spectra of the real *ε*ʹ (**a**) and imaginary *ε*″ (**b**) parts of the dielectric permittivity and a low-frequency spectrum of the absorption coefficient *α* (**c**) of Fe-doped InP. The dynamical conductivity *σ* is shown in the inset in panel (**c**). Dots are experimental data, solid lines are the modeling curves described in the main text. Dotted line in the inset corresponds to the Mott’s expression describing the hopping contribution, *σ*(ν)∝ν^s^ with *s* = 0.9. The parameters of the absorption lines (cf. Eq. (1)) are presented in Table 1.

In Table 2 we present the values of the real and imaginary parts of the dielectric permittivity, conductivity, refractive index, extinction coefficient, loss tangent tan(*δ*) = *ε*″/*ε*′ and power absorption coefficient *α* at selected frequencies. The values are listed for various frequencies *ν* between 2 cm^−1^ and 100 cm^−1^. In Fig. 3 we show the same data for the real and imaginary permittivities, absorption coefficient and dynamical conductivity *σ*(*ν*) = *νε*″(*ν*)/2 (inset) in spectral form.

**Table 2:** Dielectric parameters of the measured SI InP:Fe crystals at selected frequencies.

Wavenumber, cm^−1^	ε′ (±)	ε″ (±)	σ (±), Ohm^−1^·cm^−1^	α (±), cm^−1^	n (±)	κ (±)	Loss tangent ε″/ε′ (±)
3	12.44 (0.13)	0.01 (0.01)	0.0006 (0.0006)	0.06 (0.06)	3.527 (0.005)	0.002 (0.002)	0.001 (0.001)
6	12.43 (0.09)	0.008 (0.008)	0.0008 (0.0008)	0.08 (0.08)	3.526 (0.003)	0.001 (0.001)	0.0007 (0.0007)
10	12.42 (0.03)	0.016 (0.003)	0.003 (0.001)	0.1 (0.1)	3.524 (0.001)	0.0023 (0.0004)	0.0013 (0.0002)
20	12.43 (0.03)	0.020 (0.003)	0.007 (0.001)	0.7 (0.1)	3.526 (0.001)	0.0028 (0.0004)	0.0016 (0.0002)
30	12.42 (0.03)	0.040 (0.003)	0.020 (0.002)	2.2 (0.2)	3.524 (0.001)	0.0057 (0.0004)	0.0032 (0.0002)
40	12.43 (0.03)	0.018 (0.003)	0.012 (0.002)	1.3 (0.2)	3.526 (0.001)	0.0026 (0.0004)	0.0014 (0.0002)
50	12.47 (0.02)	0.015 (0.003)	0.013 (0.003)	1.3 (0.3)	3.531 (0.001)	0.0021 (0.0004)	0.0012 (0.0002)
60	12.52 (0.02)	0.034 (0.003)	0.034 (0.003)	3.4 (0.3)	3.538 (0.001)	0.0048 (0.0004)	0.0027 (0.0002)
70	12.55 (0.02)	0.032 (0.003)	0.037 (0.003)	4.0 (0.4)	3.5430 (0.0001)	0.0045 (0.0004)	0.0025 (0.0002)
80	12.59 (0.02)	0.060 (0.003)	0.080 (0.004)	8.6 (0.4)	3.548 (0.0001)	0.0085 (0.0004)	0.0048 (0.0002)
90	12.637 (0.002)	0.046 (0.003)	0.069 (0.004)	7.3 (0.5)	3.5550 (0.0001)	0.0065 (0.0004)	0.0036 (0.0002)
100	12.721 (0.002)	0.025 (0.003)	0.042 (0.005)	4.1 (0.5)	3.5670 (0.0001)	0.0035 (0.0004)	0.0020 (0.0002)
110	12.799 (0.002)	0.021 (0.003)	0.038 (0.006)	4.0 (0.6)	3.5776 (0.0001)	0.0029 (0.0004)	0.0016 (0.0002)
120	12.933 (0.002)	0.022 (0.003)	0.044 (0.006)	4.6 (0.6)	3.5962 (0.0001)	0.0030 (0.0004)	0.0017 (0.0002)
130	13.094 (0.002)	0.042 (0.003)	0.092 (0.006)	9.6 (0.7)	3.6186 (0.0001)	0.0058 (0.0004)	0.0032 (0.0002)
140	13.160 (0.002)	0.062 (0.003)	0.145 (0.007)	15.1 (0.7)	3.6277 (0.0001)	0.0086 (0.0004)	0.0048 (0.0002)
150	13.300 (0.002)	0.080 (0.003)	0.198 (0.007)	20.4 (0.8)	3.6469 (0.0001)	0.0110 (0.0004)	0.0060 (0.0002)
160	13.420 (0.002)	0.135 (0.003)	0.358 (0.008)	36.9 (0.8)	3.6634 (0.0001)	0.0184 (0.0004)	0.0101 (0.0002)
170	13.493 (0.002)	0.124 (0.003)	0.352 (0.008)	36.8 (0.9)	3.6733 (0.0001)	0.0169 (0.0004)	0.0092 (0.0002)
180	13.763 (0.002)	0.098 (0.003)	0.294 (0.009)	29.8 (0.9)	3.7099 (0.0001)	0.0132 (0.0004)	0.00712 (0.0002)
190	14.160 (0.002)	0.162 (0.003)	0.510 (0.009)	51.1 (0.9)	3.7630 (0.0001)	0.0216 (0.0004)	0.0115 (0.0002)
200	14.338 (0.002)	0.115 (0.003)	0.38 (0.01)	38.0 (1)	3.7866 (0.0001)	0.0152 (0.0004)	0.0081 (0.0002)

It is seen from Fig. 3 that, at THz frequencies below ≈15 cm^−1^, the measured absorption is larger than the absorption expected from impurities- or phonon-related processes (solid lines). The additional absorption at low THz frequencies should be related to the hopping conductivity of residual quasi-free charge carriers that is described by the Mott’s dispersion *σ*(ν) ∝ ν^s^ with *s* ≤ 1^27^ and was previously observed in InP crystals (see, for example ref. 28). Dotted line in the inset of Fig. 3 shows that the low-frequency contribution to the conductivity is well reproduced by the Mott’s expression with *s* = 0.9.

## Conclusion

In conclusion, we have performed room-temperature measurements of the dielectric properties of semi-insulating (SI) InP:Fe crystals in the 0.06–21 THz spectral range. Unlike the previous studies of undoped and low-doped InP material, our data unveil the dielectric properties of intrinsic InP that are not screened by the strong free-carrier absorption. A number of absorption resonances are discovered and their origin is analyzed. The values of the dielectric parameters of SI InP:Fe at frequencies between 2 and 700 cm^−1^ (0.06 and 21 THz) are presented. The data reported here is expected to be useful in designing and improving the performance of numerous microwave and terahertz semiconductor devices based on SI InP:Fe.
